# Eating Cures Dyspepsia

**Published:** 1871-07

**Authors:** 


### EATING CURES DYSPEPSIA.

Dyspepsia, or Indigestion, both which words mean essentially the same thing, is the inability of the stomach to obtain sufficient nutriment out of the food eaten to meet the wants of the system; and, not having enough, blind instinct calls for more: this call or desire for nourishment, is denominated hunger, which is gratified ordinarily by taking food into the stomach. But, in a sense, nature or instinct makes a mistake, and calls for more food when in reality it is not food that is wanting, but the nourishment which is in the food already eaten, and which the stomach has not the strength to withdraw; hence it is that a dyspeptic has a craving appetite, in aggravated cases is always eating, and is always hungry. But to eat more under such circumstances is like giving a faithful but invalid servant more work to do, when there really is not strength enough to perform what is already in hand; or it is like adding greater weight for the noble horse to draw, when he is already so oppressed by a heavy load as to be scarce able to drag it along a single step.

All dyspeptics are weak, they lack strength, the whole body is feeble, and the stomach has its share of debility, of weakness; hence the essence of cure is to increase the stomach's strength. But all bodily strength comes from the food eaten, and cannot possibly come from any other source; hence the only cure for dyspepsia is eating. But

### HOW TO EAT

Is the great practical question of this age and nation; for dyspepsia is a national disease and a national sin, since its one great cause is intemperance in eating, excessive indulgence of the appetite, in connection with unwise habits at the table.

A faithful servant may be able to do a little work well when recovering from a debilitating disease, but in the conscientious effort to perform an overtask, it is not only not accomplished, but none of it is well done. So a weak stomach may digest a little food well, get all the nourishment, all the strength out of it, but if it has to work up a large meal, the work is badly done; and as the blood is made out of the nourishment derived from the food eaten, if that nourishment is imperfect, the blood made out of it is imperfect, is bad, and all know that "bad blood" is disease.

Nor is this the only trouble: the new blood made from each meal taken is mixed in a few hours afterwards with the blood already in the system; but if this new blood is bad, it corrupts the whole mass of blood in the body, makes the whole mass of blood bad, diseased, and carries disease and discomfort to every fibre of the system; hence the ailments, the symptoms of which dyspeptics complain, are very numerous, and extend to every part of the body, — to hands, feet, head, heart, lungs, stomach, everywhere; for the hands burn after meals, the feet are cold all the time, the head aches, the heart palpitates, the lungs are oppressed, and the stomach is sick; no one dyspeptic may have all these at one time, but all and many others, in the progress of the disease, serve to make of life a protracted misery. The first great point then in the cure of dyspepsia is to

EAT BUT LITTLE AT A TIME.

And without going into detail as to other measures to be taken, it is of importance to add, that as the stomach is weak in dyspepsia, in fact is the essence of the disease, the food given it should not only be small in amount, but it should be such as is most easily worked up, most easily converted into blood; for from the blood all strength comes. As the flesh of animals, fish, poultry, is nearer being flesh of our flesh and bone of our bone than vegetables, so meat is more easily worked up by the stomach to impart nutriment to the system and make good blood than vegetables; and as bread is the staff of life, the main food of the dyspeptic should be meat and bread; the most tender meat, properly broiled, and well baked, common wheat bread, several days old, or, which is better, the whole product of the grain made up with water only, and a little salt, formed into thin, small cakes, and baked quickly in a hot oven, pan, or skillet, and eaten cold or hot.

As it requires about four hours for the stomach to digest such a meal, and it must have rest after work, just as the hands or feet require rest after their work, there should be at least five hours between the meals of dyspeptics, and not an atom of anything should be eaten between. As, therefore, there should be at least five hours' interval between meals for the dyspeptic, and it is not necessary to eat at night, for then we are asleep, it follows that we should eat not oftener than thrice a day.

But it would be of little use to get the nutriment out of the food, and make it into blood, unless it were conveyed to every part of the system, to reach every fibre, so as to impart strength to limbs and brain and stomach and lungs; to do this, exercise must be taken, for without exercise the blood begins to stagnate in half an hour, gathers around the heart, leaving the feet and hands cold and the skin chilly; and dyspeptics are always chilly and easy to take cold. And as every part of the system of the dyspeptic is weak, it is important that the exercise taken should be active enough to send the blood to the remotest parts: and as meals are taken three times a day, the exercise should be taken three times a day. And as the blood gets the greater part of its life from pure air, and there is no pure air except that out-of-doors, the exercise of the dyspeptic should be in the open air; and as exercise is more exhilarating, carries the mind more away from the body and passes time more pleasurably, it is of great importance that the exercise should be agreeable, should interest, and even absorb the whole attention; and since, taking the world as it is, the most agreeable thing to the masses is making money, that man will soonest get well of dyspepsia who steadily follows some out-door occupation which is encouragingly remunerative.

